# Epidemic Cholera; Its History, Pathology, and Treatment

**Published:** 1849-04

**Authors:** 


*Epidemic Cholera; its History, Pathology, and Treatment.* By C. B. Coventry, M. D. Professor of Obstetrics and Medical Jurisprudence in Geneva Medical College, and Professor of Physiology and Medical Jurisprudence in the University of Buffalo. Buffalo. Geo. H. Derby & Co. Publishers. 1849. 18mo. pp. 119.

This little treatise on cholera, by our respected Colleague, is opportunely submitted to the profession, and will, we think, prove an acceptable offering. Prof. C. has devoted much thought and investigation to the subject, and gives, succinctly, the views which he has been led to form, and adopt, by comparison of the opinions of others, and his own clinical study of the disease.

The appearance of the volume is highly creditable to Buffalo typography, and to the enterprising and worthy publishers, Geo. H. Derby & Co.

